# Cross-Linked Poly(vinylidene fluoride-*co*-hexafluoropropene) (PVDF-*co*-HFP) Gel Polymer Electrolyte for Flexible Li-Ion Battery Integrated with Organic Light Emitting Diode (OLED)

**DOI:** 10.3390/ma11040543

**Published:** 2018-04-02

**Authors:** Ilhwan Kim, Bong Sung Kim, Seunghoon Nam, Hoo-Jeong Lee, Ho Kyoon Chung, Sung Min Cho, Thi Hoai Thuong Luu, Seungmin Hyun, Chiwon Kang

**Affiliations:** 1 School of Advanced Materials Science and Engineering, Sungkyunkwan University (SKKU), Suwon 16419, Korea; hwan20@skku.edu (I.K.); hlee@skku.edu (H.-J.L.); 2 Department of Applied Nano Mechanics, Korea Institute of Machinery and Materials (KIMM), Daejeon 305-343, Korea; kwek14@kimm.re.kr; 3 SKKU Advanced Institute of Nanotechnology (SAINT), Sungkyunkwan University (SKKU), Suwon 16419, Korea; kbsgod2012@gmail.com (B.S.K.); hokchung@skku.edu (H.K.C.); 4 School of Chemical Engineering, Sungkyunkwan University (SKKU), Suwon 16419, Korea; sungmcho@skku.edu; 5 Center for Integrated Nanostructure Physics (CINAP), Institute for Basic Science (IBS), Suwon 16419, Korea; hoaithuongluu@gmail.com; 6 Department of Energy Science, Sungkyunkwan University (SKKU), Suwon 16419, Korea

**Keywords:** cross-linked poly(vinylidene fluoride-*co*-hexafluoropropene) (PVDF-*co*-HFP), gel polymer electrolyte (GPE), electrospinning, *N*-methyl-2-pyrrolidone (NMP), ionic conductivity, flexible Li-ion batteries, organic light emitting diode (OLED)

## Abstract

Here, we fabricate poly(vinylidene fluoride-*co*-hexafluoropropene) (PVDF-*co*-HFP) by electrospinning for a gel polymer electrolyte (GPE) for use in flexible Li-ion batteries (LIBs). As a solvent, we use *N*-methyl-2-pyrrolidone (NMP), which helps produce the cross-linked morphology of PVDF-*co*-HFP separator, owing to its low volatility. The cross-linked PVDF-*co*-HFP separator shows an uptake rate higher than that of a commercialized polypropylene (PP) separator. Moreover, the PVDF-*co*-HFP separator shows an ionic conductivity of 2.3 × 10^−3^ S/cm at room temperature, comparable with previously reported values. An LIB full-cell assembled with the PVDF-*co*-HFP-based GPE shows capacities higher than its counterpart with the commercialized PP separator, confirming that the cross-linked PVDF-*co*-HFP separator provides highly efficient ionic conducting pathways. In addition, we integrate a flexible LIB cell using the PVDF-*co*-HFP GPE with a flexible organic light emitting diode (OLED), demonstrating a fully flexible unit of LIB and OLED.

## 1. Introduction

Flexible Li-ion batteries (LIBs) have emerged as an advanced power source for flexible electronics [1]. Among key components in the flexible LIBs, the separator has served to provide ionic pathways and prevent two electrodes from being in contact for enhanced LIB performance and safety. Impregnation of liquid electrolyte into a gelable polymer separator has been a viable approach to enable a gel polymer electrolyte (GPE) for the flexible LIBs. Various polymers for separator have been studied, including: poly(vinylidene fluoride) (PVDF) [2,3], polyacrylonitrile (PAN) [4,5], poly(methylmethacrylate) (PMMA) [6], and poly(ethylene oxide) (PEO) [7]. Among these polymers, PVDF with crystallinity has gained much attention owing to its high dielectric constant (ε ≈ 8.4). It is inversely proportional to the attractive force between cation and anion of a lithium salt, which facilitates ionization of the lithium salt, thus enhancing an ionic conductivity [8,9]. Furthermore, PVDF possesses strong electron-withdrawing capability of carbon-fluorine functional groups, thus leading to electrochemical stability [10]. PVDF-based copolymers have been more commonly used than pure PVDF to fabricate a GPE possessing higher ionic conductivity [11]: PVDF-*co*-hexafluoropropene (HFP) [12,13,14,15,16,17], PVDF-*co*-chlorotrifluoroethylene (CTFE) [18], polyurethane (TPU)-PVDF [19], and PVDF-PAN [20]. In these copolymer structures, the amorphous polymer regions (e.g., HFP and CTFE) increase free spaces and polymer chain mobility for absorption of an amount of liquid electrolyte larger than pure PVDF, thus promoting lithium ion transport, while the crystalline regions (e.g., PVDF) impart their high mechanical strength to a GPE [8].

Various methods to fabricate these PVDF-based copolymer structures for a highly efficient GPE include solution casting [21,22], plasticizer extraction [23], and in-situ cross-linking [24]. Among them, electrospinning is an effective fabrication method, since electrospun fibers possess major advantages including high porosity, uniform structure, large surface area, and high permeability, leading to the high uptake of a liquid electrolyte and thus a high ionic conductivity [11,25]. Electrospun PVDF-*co*-HFP-based GPE showed the good electrochemical properties of a GPE for LIBs [2,26,27]. Recently, research teams have demonstrated PVDF-*co*-HFP-based nanocomposites with polymer and nanocrystalline ceramic fillers to improve ionic conductivity (e.g., PVDF-*co*-HFP/PAN [28], PVDF-*co*-HFP/ZnAl_2_O_4_ [29], and PVDF-*co*-HFP/PMMA/MgAl_2_O_4_ [26]). Nevertheless, few studies have been reported to engineer the morphological features for fiber structures to enhance ionic conductivity. 

Here, we demonstrated the novel structure of a cross-linked PVDF-*co*-HFP-based GPE and its implementation into a flexible LIB. We engineered the morphological features of an as-electrospun PVDF-*co*-HFP separator by employing *N*-methyl-2-pyrrolidinone (NMP), with a low volatility, as a solvent to implement a highly interconnected fiber structure and thus enhance the ionic conductivity of the GPE. With the PVDF-*co*-HFP GPE, we acquired higher capacities of a LIB cell than those of its cell counterpart with a commercialized polypropylene (PP) separator. We also integrated the battery cell with a flexible organic light emission diode (OLED) to check its possible application for flexible, mobile electronics. In a previous study, M. Koo et al. also demonstrated a flexible LIB incorporated with an OLED [30] using a glassy lithium phosphorus oxynitride (LiPON) as a solid state electrolyte. The battery performance results of this present study highlight the advantages of the PVDF-based copolymer GPEs in comparison with that of the study using LiPON, which suffers from a typically low ionic conductivity of approximately 1 × 10^−6^ S/cm at room temperature [31].

## 2. Materials and Methods

**Figure 1:**
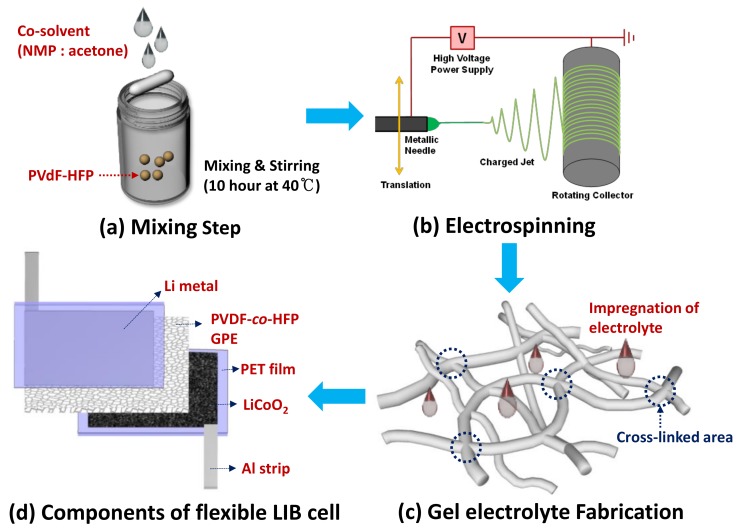
(**a**–**c**) Processing flows schematically representing the key steps to synthesize as-electrospun cross-linked poly(vinylidene fluoride-*co*-hexafluoropropene) (PVDF-*co*-HFP) fibers and their gel polymer electrolytes (GPEs) impregnated with a liquid or ionic electrolyte, and (**d**) a schematic diagram showing the key components of an assembled flexible Li-ion battery (LIB) cell integrated with the ionic-liquid impregnated PVDF-*co*-HFP fibers.

A precursor solution for a typical electrospinning method was an as-purchased copolymer poly(vinylidenefluoride)-*co*-hexafluoropropylene (PVDF-*co*-HFP) (Sigma-Aldrich, St. Louis, MO, USA) dissolved in a 16/84 (weight ratio) mixture with co-solvent of *N*-methyl-2-pyrrolidinone (NMP) (Sigma-Aldrich, St. Louis, MO, USA) and acetone (30:70 weight ratio) (Figure 1a). The solution was fed with a syringe pump and high voltage was applied between the capillary (i.e., spinneret) and grounded state stainless steel (SS) plate as a collector to fabricate randomly oriented three-dimensional (3D) electrospun PVDF-*co*-HPF fibers. Figure 1b demonstrates the schematic diagrams of the electrospinning setup and winded electrospun fibers. Table 1 summarizes the optimized parameters for the electrospinning to produce the PVDF-*co*-HFP fibers. The morphological characterizations of the as-prepared 3D PVDF-*co*-HFP fibers were tested by field emission scanning electron microscopy (FE-SEM) (SNE 4500M; SEC, Pleasanton, CA, USA). We performed dynamic mechanical analysis (DMA) of the PVDF-*co*-HFP membrane to measure its viscoelastic behavior using a dynamic mechanical analyzer (Pyris Diamond DMA; PerkinElmer, Waltham, MA, USA) at a frequency of 0.5 Hz and a ramp rate of 8 °C/min in the tension mode. 

The electrospun PVDF-*co*-HPF fibers were impregnated with a conventional liquid electrolyte [1 M lithium hexafluorophosphate (LiPF_6_) in the co-solvent of ethylene carbonate (EC) and dimethyl carbonate (DMC) (1:1 volume ratio) with an ionic conductivity of approximately 10^−2^ S/cm at room temperature] (Soulbrain, Gyeonggi-do, Korea) for a coin-type cell or ionic liquid of 1-ethyl-3-methylimidazolium bis(trifluoromethylsulfonyl)imide ([EMIM][TFSI]) with a 1 M bis(trifluoromethane)sulfonimide lithium (LiTFSI) salt (C-TRI, Gangwon-do, Korea) for a flexible-type cell (Figure 1c). In this study, we assembled a full-cell comprising a commercially available LiCoO_2_ (LCO) cathode (an areal loading density of 12 mg/cm^2^; MTI, Richmond, CA, USA) and graphite anode (an areal loading density of 8 mg/cm^2^; MTI). For a half-cell assembly, we employed Li metal as a counter and reference electrode while using LCO as a working electrode. To compare with the electrospun PVDF-*co*-HPF fibers, we employed a commercialized PP as a separator (Celgard 2400; Wellcos, Seoul, Korea). Figure 1d demonstrates the cell configuration of key components comprising a flexible LIB cell. An ionic liquid was used as an electrolyte in a flexible LIB cell owing to key physicochemical traits: high ionic conductivity, large voltage window, non-volatility, non-flammability, and excellent thermal stability [32]. To create a GPE, an as-electrospun PVDF-*co*-HFP separator was soaked in an ionic liquid [1 M lithium bis(trifluoromethylsulfonyl)imide (LiTFSI) dissolved in 1-ethyl-3-methylimidazolium bis(trifluoromethylsulfonyl)imide ([EMI][TFSI])]; subsequently, the GPE was placed in between LCO and Li metal. A flexible polyethylene terephthalate (PET) film substrate was used as a packaging material. LCO and Li metal were attached to the PET substrates through a pressure sensitive adhesion layer. Metal strips (e.g., Cu and Li) were used to connect an external circuit to a multichannel battery testing unit [(WBCS3000S; WonATech, Seoul, Korea); (CTS-Lab; Basytec, Asselfingen, Germany)] to evaluate LIB performance. The cell assembly was carried out in a dry room. Electrochemical impedance spectroscopy (EIS) measurement for lithium ionic conductivity was conducted with a symmetric cell configured by stainless steel (SS)/GPE/SS in the frequency range of 0.01–10^6^ Hz using a multi-channel potentiostat (ZIVE MP1; WonATech). Impedance results of the real and imaginary parts were plotted in the complex plane diagram (i.e., Nyquist plot) and fitted using software (Smart Manager; WonATech).

For the fabrication of organic light emitting diode (OLED), a glass substrate with size of 7 × 7 cm^2^ was cleaned with acetone, methanol, and then isopropyl alcohol for 10 min through ultra-sonication before transferring silver nanowires (Ag NWs) onto it. Hydrophobic self-assembled monolayer (SAM) was coated onto the cleaned glass to easily release the Ag NWs since a hydrophilic ultraviolet (UV) curable resin (NOA63; Norland, Cranbury, NJ, USA) was used during transfer process of the Ag NWs. For the SAM coating process, the cleaned glass was treated with a mixed solution of 500 mL toluene and 0.8 mL octadecyltrichlorosilane (ODTS; Sigma-Aldrich) for 24 h. Afterwards, the SAM on glass was cleaned with a mixed solution of toluene and ethanol (1:1 in *v*/*v*), methanol, and then acetone for 10 min in an ultra-sonication bath. The Ag NW-based solution (Nanophysis) was uniformly coated onto the SAM on glass by using a Meyer rod (#16). Subsequently, the solvent for the Ag NWs was completely evaporated at 110 °C by a heating gun, thus acquiring flat, homogeneous Ag NW layers. The Ag NWs/SAM on glass and the UV curable resin on a flexible PET film substrate were held in a “face-to-face” orientation. The assembled sample was hand-laminated for 150 s by UV irradiation to easily remove SAM on glass, thus leaving the electrode of flat-surface Ag NWs on PET. Finally, the following structured OLED was deposited onto the Ag NWs on PET electrode: HAT-CN, 40 nm (Jilin, China)/TAPC, 60 nm (Jilin, China)/SFC-H:5 wt % SFC-D, 20 nm (SFC, Cheongwon-gun, Korea)/LG-201:Liq 1:1, 30 nm (LG chem., Seoul, Korea)/Liq, 1.5 nm (OSM, Seoul, Korea)/Al (100 nm). For the stable performance of the OLED device, insulating materials were patterned through a screen printing method, whereas the OLED active area (cathode) and the area connected to the Ag NWs (anode) were protected. Lastly, the OLED on the Ag NWs coated on PET was incorporated with the assembled flexible LIB cell to demonstrate a viable way to avoid the use of a costly ITO electrode for practical, advanced flexible electronics.

## 3. Results and Discussion

**Figure 2:**
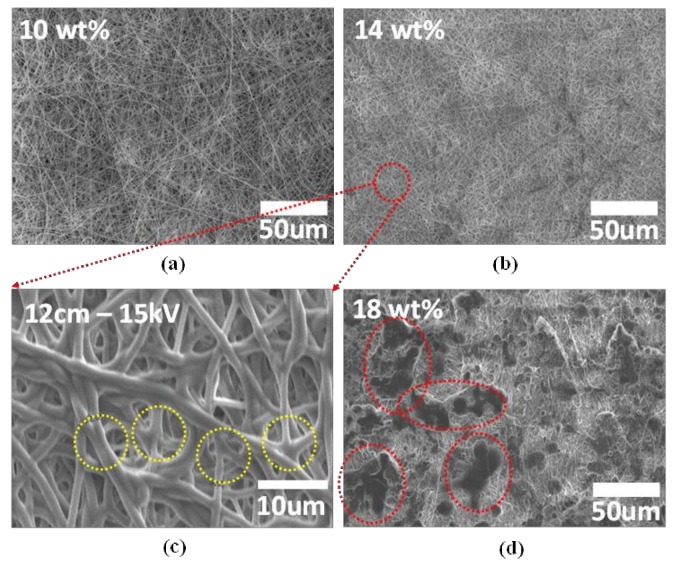
(**a**–**d**) SEM images showing the morphological variation of as-electrospun PVDF-*co*-HFP fibers with the different concentrations of the PVDF-*co*-HFP copolymer dissolved in the co-solvent of NMP and acetone. Note that (**c**) represents the magnified SEM image of (**b**) to highlight the cross-linked area marked by the yellow circles.

Figure 2 presents the morphological changes of as-electrospun PVDF-*co*-HFP fibers as a function of the concentration of PVDF-*co*-HFP dissolved in the co-solvent of *N*-methyl-2-pyrrolidinone (NMP) and acetone 3:7 (weight ratio). The overall morphology of the fibers seems nearly the same for a concentration range of 10–15 wt %. For a higher concentration, the fibers appear agglomerated as shown in Figure 2d, suggesting that solution solidification possibly occurred before jetting due to the high concentration of the copolymer.

Figure 2c is the magnified image of the PVDF-*co*-HFP fibers with an average diameter of 1.1 μm, measured by the ImageJ software (National Institutes of Health, Bethesda, MD, USA) (The conditions used for the electrospinning are summarized in Table 1). Noticeably, we observe the cross-links among different fibers (see the yellow circles), a unique feature different from the fiber morphology reported in earlier works on PVDF-*co*-HFP fibers fabricated by using solvents including *N*,*N*-dimethylacetamide (DMAc) [14,18,27] and *N*,*N*-dimethyl formamide (DMF) [3,33]. A low volatility of NMP solvent, with a low vapor pressure (3.45 × 10^−1^ mm Hg at 25 °C; 2 mm Hg at 25 °C for DMAc and 3.87 mm Hg at 25 °C for DMF), is likely to be responsible for the cross-linked fiber morphology. The NMP solvent tends to remain in the fiber networks for a longer time before evaporation than other solvents (e.g., DMAc and DMF), keeping the fibers deposited onto the grounded state plate as a collector in their molten state and thus providing a time long enough for cross-linking between the fibers. It should be noted that the cross-linked (i.e., densely interconnected) PVDF-*co*-HFP fiber network, shown in Figure 2c, suggests a possibility that, with the interconnects serving as efficient pathways for lithium ion diffusion, it could help enhance ionic conductivity [34]. Furthermore, the cross-linked and seamless networks enable high surface area between the fiber and (ionic) liquid electrolyte and a large number of interconnected pores ranging from sub-micrometer to a few micrometer in size. This structural feature could be beneficial for absorbing a large amount of (ionic) liquid electrolyte into the pores in the network structure to enhance electrolyte uptake and ionic conductivity.

**Figure 3:**
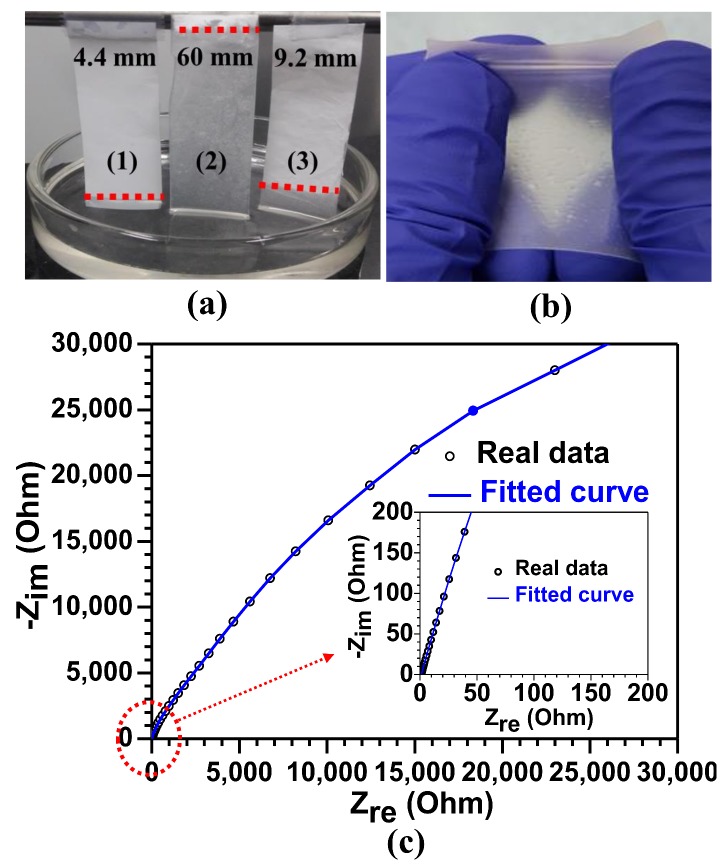
(**a**) Comparative investigation on the absorbed levels of liquid electrolyte into (1) commercially available polypropylene (PP) separator and as-electrospun cross-linked PVDF-*co*-HFP separators with the two different thicknesses of (2) 40 μm and (3) 100 μm for 10 s, (**b**) a photo image representing a structural robust and stretchable PVDF-*co*-HFP GPE encapsulating 1 M LiPF_6_ in EC: DMC, and (**c**) electrochemical impedance spectra (EIS) results of the PVDF-*co*-HFP GPE used in the stainless steel (SS)/GPE/SS cell.

Next, we immersed the separators of the PVDF-*co*-HFP fibers with two different thicknesses (i.e., 60 and 100 μm) in liquid electrolyte [1 M LiPF_6_ in EC and DMC (1:1 volume ratio)] for around 10 s to check electrolyte absorption. A commercially available PP-based separator was immersed together for comparison. The fiber-based separators showed much more rapid absorption: for the 100-μm-thick PVDF-*co*-HFP fiber separator, the uptake rate was measured to be 6 mm/s, which is approximately 13-fold higher than that of the PP-based separator (0.44 mm/s), as shown in Figure 3a. The higher uptake rate of the PVDF-*co*-HFP fibers could be attributed to the efficient lithium ion diffusion pathways through the cross-linked PVDF-*co*-HFP network, together with its good wettability and compatibility (or affinity) with the liquid electrolyte. Moreover, Figure 3b is a photo image representing a flexible PVDF-*co*-HFP GPE after removing an excess electrolyte. Note that the GPE retains its structural integrity and mechanical strength after its liquid electrolyte uptake and structural deformation. We employed dynamic mechanical analysis (DMA) of the PVDF-*co*-HFP membrane to investigate its viscoelastic behavior (see Figure S1 in the Supplementary Information), thus confirming that the storage modulus (G′) of the membrane decreases with the temperature ranging from 20 to 156 °C, rendering the membrane stretchable [35].

Figure 3c reveals the Nyquist plot of a cell configured with SS/PVDF-*co*-HFP GPE/SS to measure an ionic conductivity of the PVDF-*co*-HFP GPE in the cell. The bulk resistance (R_b_) is equivalent to the intercept point in the real part of resistance (i.e., at imaginary part of resistance (*Z*″ = 0)) in the Nyquist plot. From this value, the ionic conductivity is measured to be 2.3 × 10^−3^ S/cm at room temperature, which is compared with those of previous reports [2,26,27] and sufficient for practical LIB [5]. The high ionic conductivity mainly originates from a high amount of the liquid electrolyte impregnated in the pore networks of the PVDF-*co*-HFP separator. As mentioned earlier, the cross-linked PVDF-*co*-HFP network structures can contain a large amount of liquid electrolyte owing to their effective enclosure. Furthermore, amorphous HFP copolymer regions absorb the liquid electrolyte and enable crystalline PVDF regions to promote the ionization of the LiTFSI salt to increase the concentration of Li^+^ ions, thus enhancing a lithium ionic conductivity [8]. In this sense, the amorphous and swollen HFP regions caused by the absorption of a liquid electrolyte can offer lithium ionic conducting pathways through the cross-linked network structures [8].

**Figure 4:**
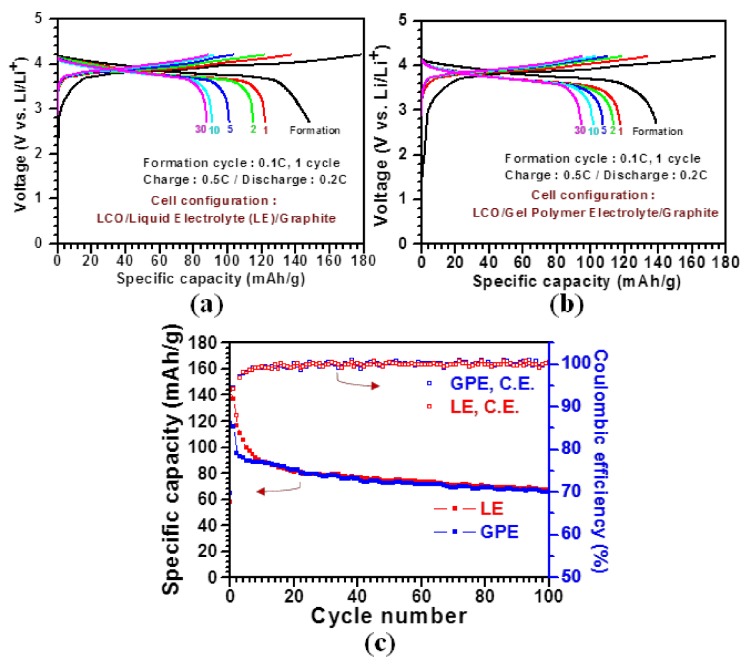
A comparative study of the performance of LIB cells incorporated with the commercialized PP and the PVDF-*co*-HFP separators. (**a**,**b**) Galvanostatic charge and discharge profiles of the LIB full-cells with the two different separators at 0.5 C for 30 cycles, and (**c**) cycling performance of the LIB half-cells with the two different separators at 0.5 C for 100 cycles and their corresponding coulombic efficiencies.

Figure 4a,b compare LIB performances between the commercialized PP and the PVDF-*co*-HFP separators by testing full-cells with LiCoO_2_ (LCO) and graphite used as cathode and anode materials, respectively. Note that the PVDF-*co*-HFP separator-based cell delivers a specific capacity of ~94 mAh/g after 30 cycles, which is approximately 8% higher than that (~87 mAh/g) of the PP separator-based cell. This capacity enhancement is closely ascribed to the unique cross-linked GPE structure.

Furthermore, we compared LIB performances between the PP and the PVDF-*co*-HFP separators by testing half-cells with LCO and Li metal as a working electrode, and a counter and reference electrode, respectively, in the voltage window from 3 to 4.5 V at 1 C for 100 cycles (Figure 4c). The PVDF-*co*-HFP GPE-based cell shows a specific capacity of 65 mAh/g after 100 cycles, which is approximately 2% lower than that of the PP-based cell (67 mAh/g). Moreover, the coulombic efficiencies of both cells are recorded to be nearly 100% after 3 cycles. With these LIB performances, we confirm that the cross-linked PVDF-*co*-HFP GPE could be implemented into practical LIB cells as a substitute for the conventional PP-based separator. 

**Figure 5:**
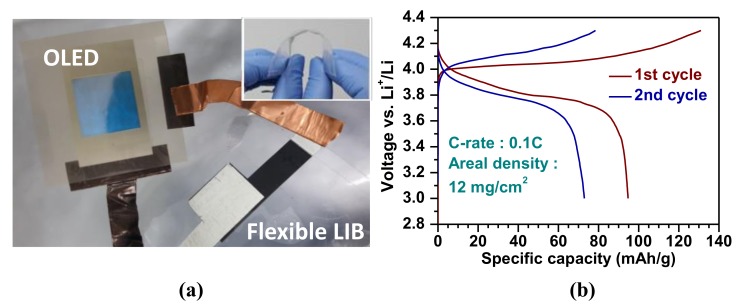
(**a**) A photograph of the assembled flexible LIB cell illuminating a blue OLED under operation at approximately 4 V. The inset image demonstrates the flexible LIB cell under bending condition illustrating the cell’s thinness (<1 mm) and structural flexibility. (**b**) Initial galvanostatic charge and discharge profiles of the flexible LIB cell with the ionic liquid impregnated GPE at 0.1 C.

In this study, we integrated our flexible LIB cell with a flexible OLED to further test it for its possible application for flexible and mobile electronics, as shown in Figure 5. Figure 5a represents a photo image of a blue OLED powered by the assembled flexible LIB cell (see a video in Supplementary Materials for the operation of the OLED connected to the flexible LIB). Note that the inset image illustrates the high flexibility of the LIB cell under bending condition. Charge-discharge cycles were conducted by galvanostatic mode (constant applied current density) with the voltage window of 3–4.3 V at 0.1 C for 2 cycles (Figure 5b). The specific capacity for the 1st cycle is around 95 mAh/g and significantly drops to 73 mAh/g for the 2nd cycle. The drastic drop in capacity probably arose from packaging issues of the LIB cell, such as incomplete sealing, which could be resolved by employing a commercial packaging process (for aluminum pouch cell).

Our capacity data (1140 µAh/cm^2^ for the 1st cycle and 876 µAh/cm^2^ for the 2nd cycle in areal capacities calculated using the LCO loading density of 12 mg/cm^2^) compares well with the value (~110 µAh/cm^2^) reported in a previous approach for a flexible LIB integration with a flexible OLED using a LiPON solid state electrolyte [30]. The significant improvement of the capacity shown in this present study is believed to arise from the high ionic conductivity (2.3 × 10^−3^ S/cm, much higher than that of LiPON, ~1.0 × 10^−6^ S/cm at room temperature) of the GPE and the high areal loading density of the active material [31]. The difference in ionic conductivity is believed to mainly come from two aspects: (1) difference in ionic conductivity between the solid state electrolyte and PVDF-*co*-HFP GPE, (2) the cross-linked network design of our GPE. 

In this study, we fabricated the cross-linked PVDF-*co*-HFP fibers as a separator using electrospinning for the high-performance GPE. The cross-linked PVDF-*co*-HFP-based GPE showed a high ionic conductivity, thus enabling its incorporated LIB cell to acquire a capacity higher than that of the commercialized PP separator-based cell. We further integrated the cell with the flexible OLED: although the cycle stability was found poor due to packaging issues, the capacities of the 1st and 2nd cycles were found high, highlighting the possibility that further engineering the morphology of electrospun fibers makes GPE available for flexible and mobile electronics.

## 4. Conclusions

In this study, we fabricated the electrospun, cross-linked PVDF-*co*-HFP-based GPE for the advanced flexible LIB. NMP solvent, with a low volatility, played a primary role in producing the cross-linked morphology of the fibers, which led to a high uptake capability of electrolyte through the formation of the well-interconnected pore structures. Furthermore, the PVDF-*co*-HFP separator exhibited an ionic conductivity of 2.3 × 10^−3^ S/cm at room temperature, which is comparable with the previously reported values and sufficient for practical LIB applications. Moreover, the LIB full-cell assembled with the PVDF-*co*-HFP-based GPE demonstrated a capacity superior to its cell counterpart with the commercialized PP-based separator. Lastly, we implemented the PVDF-*co*-HFP GPE into a flexible LIB cell in integration with the flexible OLED, demonstrating the fully flexible unit of LIB and OLED.

## Figures and Tables

**Table 1:** Key optimized parameters for the electrospinning to fabricate the cross-linked PVDF-*co*-HFP fiber networks.

Parameters	Values
Copolymer concentration in solution	14–16 wt %
Spinning solution volume	9 mL
Spinning rate	3 mL/h
Spinning time	3 h
Working distance between the capillary and the collector	15 cm
Electrical potential	17 kV
The rotation frequency of the collector	100 rpm
Width of as-electrospun fiber sheet	110–150 cm

## Supplementary Materials

The following is available online at http://www.mdpi.com/1996-1944/11/4/543/s1. Video S1: A video clip for the operation of the OLED connected to a flexible LIB and Figure S1 illustrating the dynamic mechanical analysis (DMA) results of the PVDF-*co*-HFP membrane.
