# Supplementary material for: Cross-Linked Poly(vinylidene fluoride-co-hexafluoropropene) (PVDF-co-HFP) Gel Polymer Electrolyte for Flexible Li-Ion Battery Integrated with Organic Light Emitting Diode (OLED)
Source: Materials (Basel). 2018 Apr 2;11(4):543. doi: 10.3390/ma11040543 (PMC5951427; doi:10.3390/ma11040543)
Supplement: Supplementary file 1 [file materials-11-00543-s001.zip › Supplementary Information for Materials-Final.docx]

Supplementary Information

Cross-Linked Poly(vinylidene fluoride-*co*-hexafluoropropene) (PVDF-*co*-HFP) Gel Polymer Electrolyte for Flexible Li-Ion Battery Integrated with Organic Light Emitting Diode (OLED)

Ilhwan Kim ^1,3,†^, Bong Sung Kim ^2,†^, Seunghoon Nam ^3^, Hoo-Jeong Lee ^1^, Ho Kyoon Chung ^2^,
Sung Min Cho ^4^, Thi Hoai Thuong Luu ^5,6^, Seungmin Hyun ^3,^* and Chiwon Kang ^1,^*

**Figure S1.** The variation in the storage and loss modulus, and tangent loss of the PVDF-*co*-HFP membrane as a function of temperature.
